# Novel Morphological Profiling Assay Connects *ex Vivo* Endothelial Cell Responses to Disease Severity in Liver Cirrhosis

**DOI:** 10.1016/j.gastha.2023.10.006

**Published:** 2023-10-24

**Authors:** Rudmer J. Postma, Annelotte G.C. Broekhoven, Hein W. Verspaget, Hetty de Boer, Thomas Hankemeier, Minneke J. Coenraad, Vincent van Duinen, Anton Jan van Zonneveld

**Affiliations:** 1 Department of Internal Medicine (Nephrology) and the Einthoven Laboratory for Vascular and Regenerative Medicine, Leiden University Medical Center, Leiden, The Netherlands; 2 Department of Gastroenterology and Hepatology, Leiden University Medical Center, Leiden, The Netherlands; 3 Department of Analytical BioSciences, Leiden Academic Centre for Drug Research, Leiden University, Leiden, The Netherlands; 4 MIMETAS B.V., Oegstgeest, The Netherlands

**Keywords:** Endothelial Cell Dysfunction, Liver Cirrhosis, Morphological Profiling, Mitochondria

## Abstract

**Background and Aims:**

Endothelial cell (EC) dysfunction in response to circulating plasma factors is a known causal factor in many systemic diseases. However, no appropriate assay is available to investigate this causality *ex vivo*. In liver cirrhosis, systemic inflammation is identified as central mechanism in progression from compensated to decompensated cirrhosis (DC), but the role of ECs therein is unknown. We aimed to develop a novel *ex vivo* assay for assessing EC responses to patient-derived plasma (PDP) and assess the potential of this assay in a cohort of liver cirrhosis patients.

**Methods:**

Image-based morphological profiling was utilized to assess the impact of PDP on cultured ECs. Endothelial cell (EC) monolayers were exposed to 25% stabilized PDP (20 compensated cirrhoses, 20 DCs, and 20 healthy controls (HCs). Single-cell morphological profiles were extracted by automated image-analysis following staining of multiple cellular components and high-content imaging. Patient profiles were created by dimension reduction and cell-to-patient data aggregation, followed by multivariate-analysis to stratify patients and identify discriminating features.

**Results:**

Patient-derived plasma (PDP) exposure induced profound changes in EC morphology, displaying clear differences between controls and DC patients. Compensated cirrhosis patients showed overlap with healthy controls and DC patients. Supervised analysis showed Child-Pugh (CP) class could be predicted from EC morphology. Most importantly, CP-C patients displayed distinct EC phenotypes, in which mitochondrial changes were most discriminative.

**Conclusion:**

Morphological profiling presents a viable tool to assess the endothelium *ex vivo*. We demonstrated that the EC phenotype corresponds with disease severity in liver cirrhosis. Moreover, our results suggest the presence of mitochondrial dysfunction in ECs of CP-C patient.

## Graphical abstract

**Figure:**
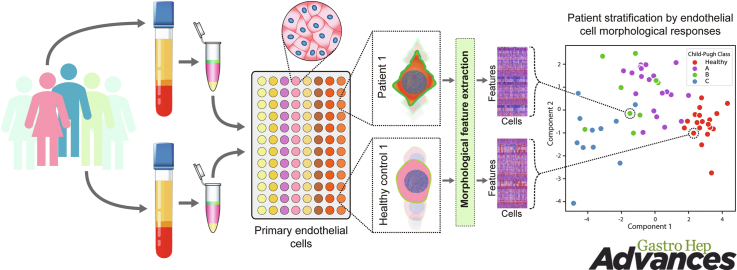

## Introduction

The endothelium forms the inner lining of the blood vessels. It plays a central role in many physiological processes, including transport of cells and solutes, hemostasis, control of vascular permeability and regulation of vascular tone.^1^ In addition, endothelial cells (ECs) play a key role in regulating the innate immune response.^2^^,^^3^ In a noninflamed state, ECs display a quiescent phenotype, while in response to inflammatory mediators, ECs readily assume an activated state that is associated with a variety of morphological changes, including expression of adhesion molecules and actin cytoskeleton reorganization.^4^ Patients with systemic chronic inflammation can develop EC dysfunction, a pathological state of the endothelium implicated in a variety of medical conditions, including sepsis, cardiovascular diseases, diabetes, systemic sclerosis, chronic kidney disease, and has also been identified as a key driver of SARS-CoV2-related complications.^2^^,^^5^, ^6^, ^7^, ^8^, ^9^

Liver cirrhosis is an end-stage liver disease with different etiologies such as viral hepatitis or alcohol-related disease. The clinical course of liver cirrhosis can be divided into 2 stages: a compensated, asymptomatic phase and a decompensated symptomatic phase. Once decompensation has occurred, cirrhosis becomes a systemic disease, with multiorgan/system dysfunction.^10^ Manifestations of decompensated cirrhosis are ascites, variceal bleeding and hepatic encephalopathy.^10^ Liver cirrhosis is also characterized by major circulatory disturbances in both the intrahepatic and systemic vascular systems.^11^^,^^12^

There are 2 main pathophysiological mechanisms underlying decompensated cirrhosis: systemic inflammation and portal hypertension. Systemic inflammation is correlated with the progression from a compensated into a decompensated state.^12^^,^^13^ This systemic inflammation is hypothesized to result from changes in the gut microbiome and bacterial overgrowth, leading to an altered intestinal permeability which facilitates translocation of bacteria and their metabolites into the portal and systemic circulation.^14^, ^15^, ^16^ There, bacterial components such as lipopolysaccharides (LPS), and tumor necrosis factor alpha (TNF-α) produced by mononuclear cells, induce the proinflammatory response, which eventually can lead to EC dysfunction and result in organ failure.^17^^,^^18^ As a consequence of the sustained systemic inflammation, oxidative stress in the intrahepatic circulation is increased and nitric oxide production by the liver sinusoidal EC is decreased, resulting in increased intrahepatic resistance and portal hypertension.^11^^,^^19^ In contrast, the systemic circulation in decompensated cirrhosis displays nitric oxide overproduction with peripheral arterial vasodilation, mainly in the splanchnic area, leading to reduced effective circulating volume and peripheral organ hypoperfusion, which may favor the development of organ dysfunction or failure.^11^

Decompensated cirrhosis is associated with high 1-year mortality rates, ranging from 10% to 75% depending on the clinical course.^20^ Importantly, it has been shown that prognosis of decompensated patients is correlated to the degree of systemic inflammation.^13^

We hypothesize that disease progression is reflected in the state and phenotype of the endothelium in systemic diseases such as liver cirrhosis. However, assessing the endothelium directly poses a significant challenge for clinicians and researchers as no laboratory tests are available.^21^ Since cultured primary ECs respond to inflammatory mediators and endotoxins by morphological changes, we aim to demonstrate that profiling EC morphological responses to individual patient-derived plasmas reflects disease progression.

In a recent study, we demonstrated that disease-specific endothelial phenotypes can be induced *ex vivo* by exposing ECs to patient-derived stabilized and recalcified dipotassium ethylenediaminetetraacetic acid (EDTA) plasma *in vitro*.^22^ Moreover, our approach allowed for prolonged exposure to EDTA plasma, thereby allowing for sufficient time for ECs to change their phenotype. However, this methodology employed a metabolomics readout, which is medium-throughput only, time-consuming, and costly. Developments in high-throughput, high-content image-based screenings (HCS) and data analysis methods have resulted in HCS to become the industry standard in characterizing cellular phenotypes in small-molecule screening.^23^^,^^24^ State-of-the-art HCS-based assays, such as the “Cell painting assay,” developed by Bray et al.,^25^ allows one to obtain a broad overview of the cellular states and phenotypes present in a sample by computing single-cell morphological profiles from a multiplex of microscopy images.^23^^,^^25^ Therefore, HCS may be suitable to detect EC phenotypical responses to patient-derived plasma, as ECs will assume different morphologies depending on the integration of plasma signals.^26^, ^27^, ^28^

We present the design and validation of a high-throughput, high-content image-based assay for the assessment of EC morphological responses to patient-derived plasma. We demonstrate that our assay allows the stratification of disease progression in liver cirrhosis and may well provide a valuable translational tool to dissect the complex circulating risk factors in diseases in which systemic inflammation is involved in general.

## Materials and Methods

### Patient Cohort

Cases were selected from a local biobank database consisting of patients with liver diseases (approval for the Liver Diseases Biobank by the Medical Ethics Committee MDL 064/SH/sh;3.4120/09/FB/jr). All patients gave written informed consent for inclusion in the biobank. EDTA samples from 2 different patient groups were selected: (i) patients with compensated cirrhosis and (ii) patients with decompensated cirrhosis. Diagnosis of cirrhosis was based on histology, imaging and/or laboratory results. Compensated cirrhosis was defined as having a Child-Pugh (CP) class A with no overt symptoms indicative of decompensation and without a history of decompensation.^29^ Decompensated cirrhosis was defined as having a CP class B or C with one or more of the following symptoms: hepatic encephalopathy, variceal bleeding, or clinically manifested ascites (ie ≥ grade 2 according to the international Ascites Club).^10^^,^^30^ Exclusion criteria for both groups were diagnosis of an underlying liver disease of primary sclerosing cholangitis with recurrent cholangitis, a history of any malignancy, diagnosis of any malignancy within 1 year after sample retrieval, any active systemic autoimmune disease. Electronical medical records of all included patients were retrospectively reviewed to collect clinical data. Information was collected on etiology of underlying liver disease, comorbidities, complications of cirrhosis, presence of infection, laboratory values, severity scorers (i.e. CP score,^31^ model of end-stage liver disease [MELD] score^32^) and medication use. Infection was defined as having a polymorphonuclear cell count in ascites fluid ≥ 250/mm^3^; having a positive blood culture; having a positive urinary culture; having a positive ascitic fluid culture; having lesions on chest radiography suspected for pneumonia).

Healthy controls (HCs) were included via a separate local biobank protocol established for healthy subjects, named “the Leiden University Medical Center Volunteer Donor Service biobank”. All HCs gave written informed consent. From these controls, data only on age, gender and health status are available. The study protocol conformed to the ethical guidelines of the 1975 Declaration of Helsinki and approval was obtained by a local ethics committee (METC LDD number B21.014).

### Patient Characteristics

Patients with compensated (n = 20) and decompensated (n = 20) cirrhosis were included in the study. Characteristics of these groups are summarized in Table. Patients in the decompensated cirrhosis group tended to be older (mean 57 vs 65 years) and more frequently male (45% vs 70%), but differences were not statistically significant. Moreover, no significant differences in etiology of cirrhosis were observed between the groups. Decompensated patients were more severely ill (ie higher serum bilirubin concentration, lower serum creatinine concentration, and a higher International Normalized Ratio) and had a higher MELD score (median 17 (interquartile range (IQR) 14.8–20.3) vs 7 (IQR 7–9), *P* < .001). Within the decompensated group, 9 (45%) patients were diagnosed with CP class B, and 11 (55%) with CP class C. One patient was diagnosed with an infection (cholangitis) at time of the sample collection. No differences in presence of comorbidities were observed between the 2 groups. Forty-five percent of the patients in the decompensated group received antibiotic therapy, whereas none of the patients in the compensated group received antibiotics (*P* = .005). Other therapies such as immunosuppressive therapy or albumin did not differ between the 2 groups.

**Table:** Patient characteristics

Characteristic	Compensated (n = 20)	Decompensated (n = 20)	*P* value
Age, median (IQR)	57 (50.5–64.5)	65 (52.75–68)	.189
Gender			.201
Male, n (%)	9 (45)	14 (70)	
Female, n (%)	11 (55)	6 (30)	
Aetiology liver disease, n (%)			.057
Alcoholic liver disease	1 (5)	9 (45)	
Viral hepatitis	4 (20)	2 (10)	
AIH	7 (35)	3 (15)	
NASH	3 (15)	3 (15)	
Other	5 (25)	3 (15)	
Portal hypertension, n (%)	0 (0)	3 (15)	.072
Ascites, n (%)	0 (0)	18 (90)	<.001
Hepatic encephalopathy, n (%)	1 (5)	8 (40)	.043
Infection, n (%)	0 (0)	1 (5)	1.000
Creatinine, median (IQR)	64 (54–71)	74 (56.5–90.5)	.093
Bilirubin, median (IQR)	16 (10–31)	111 (34.75–130.75)	<.001
INR, median (IQR)	1.1 (1.0–1.1)	1.5 (1.4–1.7)	<.001
WBC	4.87 (3.75–7.21)	5.81 (2.4–7.22)	.939
Child Pugh class, n (%)			<.001
A	20 (100)	0 (0)	
B	0 (0)	9 (45)	
C	0 (0)	11 (55)	
MELD score, median (IQR)	7 (7–9)	17.5 (14.75–20.25)	<.001
CLIF-AD score, mean ± SD	38.31 (5.52)	50.19 (9.78)	<.001
ACLF, n (%)	0 (0)	2 (10)	.468
Comorbidities, n (%)			
Hypertension	6 (30)	7 (35)	1.000
Cardiovascular disease	1 (5)	4 (20)	.442
Chronic kidney disease	0 (0)	1 (5)	1.000
Diabetes	5 (25)	6 (30)	1.000
Medication			
Immunosuppression	5 (25)	1 (5)	.119
Corticosteroids	6 (30)	3 (15)	.294
Antibiotics	0 (0)	9 (45)	.005
Statins	3 (15)	2 (10)	.845
Albumin	0 (0)	3 (15)	.288

ACLF, acute-on-chronic liver failure; AIH, autoimmune hepatitis; CLIF-AD, CLIF Consortium Acute Decompensation score; NASH, nonalcoholic steatohepatitis; WBC, white blood cell count.

In the HC group, 20 subjects were included. Ten (50%) subjects within this group were male, and the median age was 32 years (IQR 28–39.75). The age between the HCs and the compensated and decompensated group differed significantly (*P* < .001). No difference in gender inclusion was observed (*P* = .243).

### Lipopolysaccharide (LPS) Measurements

Lipopolysaccharide (LPS) concentrations were measured using a chromogenic kit (Pierce Chromogenic Quant Kit, ThermoFisher A39552), according to the manufacturer’s instructions. In short, plasma samples were diluted 1:50 in endotoxin free water, heat-inactivated at 80 °C for 15 minutes, and incubated with reagents. Absorption units were used for further analysis instead of concentrations, as plasma components interfered with absolute quantification.

### Cell Culture

Human umbilical vein endothelial cells (HUVECs) were isolated from umbilical cords obtained from the department of obstetrics at the Leiden University Medical Center, as previously described.^33^ Freshly isolated HUVECs were cultured in Endothelial cell growth medium 2 (EGM-2) (PromoCell C-22111) supplemented with 1% Penicillin/Streptomycin (Gibco 15070-063) for one passage and cryogenically frozen.

HUVECs were thawed and cultured in a 1% gelatin (Merck; 104078) coated T75 flask 2 days before seeding in the 96-wells plate (Corning 4580). Glass surfaces of the 96-wells plate were coated according to Araica et al.^34^ In short, wells were incubated overnight with 0.5% glutaraldehyde (Sigma Aldrich; G 400-4) in water (Ampuwa), washed, and incubated with water overnight. Before seeding, wells were incubated with 1% (w/v) gelatin in phosphate-buffered saline (PBS) (Gibco 14190-094) for 30 minutes at 37 °C, followed by incubation with 0.5% glutaraldehyde in PBS for 20 minutes. Wells were washed repeatedly with PBS, incubated with 1% Glycine (Merck 1.04201) in PBS for 7 minutes, followed by incubation with PBS for at least 4 hours before seeding the cells. HUVECs were seeded at a density of 9000 cells/well in 100μL EGM-2 medium. Cells were cultured for 2 days before plasma exposure to allow a cobblestone-like monolayer to form.

### Tumor Necrosis Factor Alpha (TNF-α) and Lipopolysaccharide (LPS) Stimulation

Confluent HUVEC monolayers were exposed to either 0, 0.010, 0.10, 1.0 ng/ml TNF-α (Sigma-Aldrich H8916) or 0, 0.020, 0.20, 2.0, 20, 200, 2000 ng/ml LPS from e. Coli (Enzo Life Sciences ALX-581-010-L001) in 25% HC plasma for 18 hours. Five replicate wells were exposed per condition.

### *Ex Vivo* Plasma Exposure

Low passage (*P* = 2), single-donor, confluent HUVEC monolayers were exposed to 25% recalcified K_2_EDTA plasma derived from either patients or HCs for 18 hours. K_2_EDTA plasma was stabilized and recalcified by adding 0.5 μM recombinant Hirudin (ABCAM ab201396), 25 μg/ml Corn trypsin inhibitor (HTI CTI-01), 12.5 μM Compstatin (kind gift from dr John Lambris, University of Pennsylvania, USA) and 1.85 mM calcium-dichloride (CaCl_2_) (Merck 1.02381) to a K_2_EDTA plasma volume equaling 25% of the total volume.^22^ Endothelial cell basal medium (PromoCell C-22211) containing 1:100 insulin transferrin selenium supplement (Gibco 41400) was added to obtain the final volume. Samples were equally distributed between plates according to age, sex, and disease severity and subsequently randomly distributed across the individual plate. Three replicate wells were exposed for each plasma sample.

### Immunofluorescent Staining and Imaging

Before fixation, cells were incubated with MitoTracker Deep Red FM (InVitrogen M22426) 1:2000 for 30 minutes. Cells were fixed by incubation with 3.6% paraformaldehyde (Alfa Aesar J61899) + 0.5% (w/v) albumin from bovine serum (BSA) (Sigma A7030) in PBS for 15 minutes, washed with blocking buffer (5% (w/v) BSA in HBSS+ (Gibco 14025-050)), and permeabilized by incubation with 0.2% (v/v) Triton X-100 in HBSS + for 10 minutes. Afterwards, cells were incubated with blocking buffer for 10 minutes, followed by 2-hour-long incubation with primary antibody against vascular endothelial cadherin (VE-Cadherin) (BD 555661, 2μg/ml in HBSS+ + 0.5% (w/v) BSA). Cells were washed 3 times with blocking buffer and incubated with 488 Alexa-fluorophore labeled Donkey antimouse antibody (Invitrogen A-11001, 2 μg/ml), 1:200 Rhodamine Phalloidin (Invitrogen R415), and 1:1000 HOECHST 33258 (Molecular Probes) in HBSS+ with 0.5% BSA for 1 hour. Cells were washed with blocking buffer and stored under HBSS+.

Max-projections of 15 z-steps with 0.6 μm step size were acquired using a high content confocal microscope (Molecular Devices, ImageXpress™ Micro Confocal) at 20x magnification (Nikon Plan Apo Lambda; NA = 0.75). Dapi, FITC, TRITC, and CY5 channels were used to acquire nuclei, VE-Cadherin, F-actin, and mitochondria, respectively. Eight sites without overlap were imaged per well.

### Image Quality Control and Preprocessing

For each image, a focus-score metric,^25^^,^^35^ intensity sum, median, standard deviation, and the 0.01, 0.25, 0.75, 0.99 quantiles were computed. Results were plotted in histograms comprised of all images for a given channel to assess outliers.^25^ Visual inspection of outliers was performed to assess image quality before discarding. Images that contained artifacts (eg out-of-focus images, debris, clipping/saturation artifacts) were discarded. Sites that contained failed images in a single channel were discarded altogether. Following image quality control, images were corrected for uneven illumination and vignetting, based on the iterative approach by Draper et al.^36^

### Image Analysis

Single-cell morphological profiles were extracted from the illumination corrected images using R (version 4.1.1),^37^ the R-package EBImage (version 4.29.2).^38^ In short, individual nuclei were identified from Dapi-channel images by first applying a local threshold, followed by applying watershed segmentation to identify individual nuclei. Debris was filtered out by applying a lower threshold for nuclei size. Cells were identified by Voronoi-Based segmentation using the VE-Cadherin signal to identify cell borders, using individual nuclei as seeds. R-package EBImage “compute” functions were used to compute shape, moment, intensity, and texture image-based features. Functions to extract distribution and colocalization were written in-house, based on the procedure by Verbeek et al.^39^

### Data Analysis

Data analysis concerning patient data was performed using R(version 4.1.1.) Qualitative data are described using frequencies and percentages. Normality of the distribution was preassessed according to Kolmogorov–Smirnov test. Quantitative variables are described using mean (standard deviation) or median (IQR) when appropriate. Comparisons between independent groups were performed using the Mann–Whitney U-test and t-test for continuous variables, and Chi-square test or Fisher’s exact test for categorical variables. A *P* value < .05 was considered to be statistically significant.

Data analysis concerning the assay was performed in Python version 3.7.6, using functions from the Scikit-learn package.^40^ The data analysis approach was as follows: Z-score normalization was applied to each plate dataset separately.^25^ Afterwards, datasets were combined. Single-cell profiles were reduced in dimension by factor analysis,^41^ capturing about 80% of total variance. Mean profiles were created by averaging the cells per well for each technical replicate, condition, or patient in the reduced subset. Resulting profiles were scaled to 0-mean and unit variance before analysis. For supervised analysis, linear discriminant analysis (LDA) or partial least squares (PLS) models were constructed. Cross-validation was performed by repeatedly leaving out 10% or 20% of the data before data scaling and model fitting. Categorical or numerical values of the left-out subset were predicted by feeding the left-out subset into a fitted standard scaler and LDA or PLS model, respectively.

## Results

### Development of Assay Workflow

**Figure 1:**
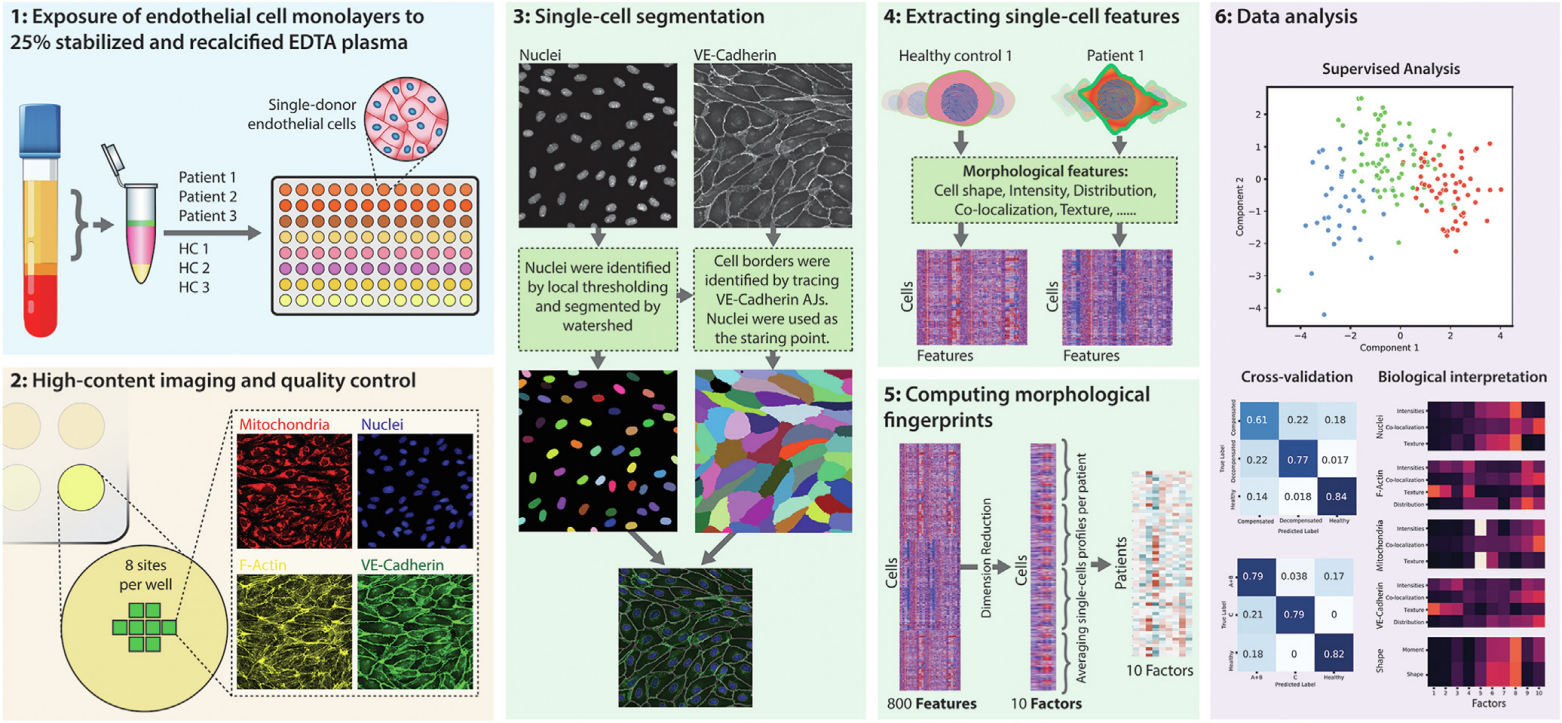
Overview of the workflow of the *ex vivo* endothelial cell morphological assay. 1: Patient or healthy control plasma was stabilized by adding coagulation and complement inhibitors, and recalcified by adding calcium chloride. Basal medium was added to reach a final 25% plasma concentration. Subsequently, single-donor endothelial cell monolayers were exposed to 25% recalcified plasma mix and incubated for 18 hours. 2: Mitochondrial staining was performed before fixation by incubating with MitoTracker deep-red FM for half an hour. After fixation, cells were stained for nuclei, f-actin, and VE-cadherin. Afterwards, 8 sites per well were imaged using a high NA 20× objective lens. Image quality control was performed and images containing artifacts were discarded. 3: Nuclei were segmented by first applying a local threshold, followed by separation into individual nuclei by the watershed algorithm. Subsequently, nuclei were used as starter seeds for identification of the cell borders by Voronoi tessellation. 4: Morphological features were extracted at the single-cell level using the previously determined segmentation. These features included cell shape, intensity of the staining, distribution of the staining within the cell, colocalization, and texture. This procedure resulted in a dataset containing an 800-dimensional vector for each cell. 5: All single-cell profiles were combined into one dataset, and dimension reduction was performed using Factor Analysis. The reduced dataset contained around 10 factors, capturing 80% of total variance. Single morphological fingerprints were computed for each patient by averaging all singe-cell data from each patient, resulting in 1 vector per patient with the length of the total number of Factors. 6: Data analysis of the resulting profiles was performed using PCA and LDA. Biological interpretation of the results was aided by investigating factor contrition to the clustering observed in the PCA or LDA score plots.

A detailed overview of the array workflow is visualized in Figure 1. In short, cultured EC monolayers were exposed to 25% stabilized and recalcified EDTA plasma for 18 hours to elicit morphological responses. This 25% plasma concentration was chosen as it was the highest plasma concentration possible without causing excess cellular stress in ECs exposed to healthy donor plasma (Figure A1). Subsequently, morphological fingerprints were computed for each patient or healthy control by automated image-analysis on a single-cell level followed by dimension reduction and averaging. The resulting patient-specific morphological fingerprints were used for patient stratification and further data analysis.

### Validation of Assay Performance

**Figure 2:**
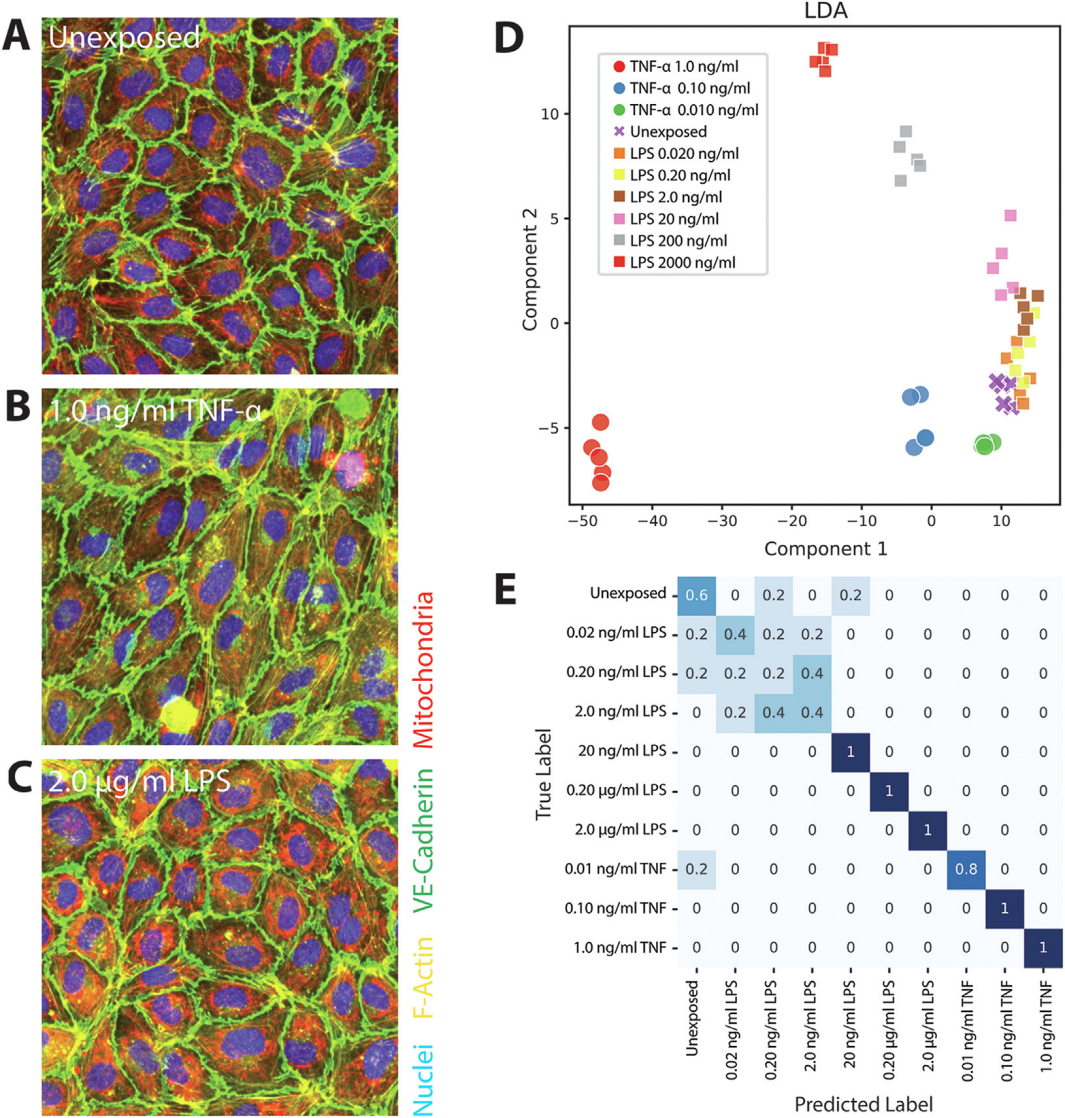
Supervised analysis of TNF-α and LPS stimulation. (A–C) Examples of distinct cell morphology of endothelial cell monolayers, nonexposed (A) exposed to 1.0 ng/ml TNF-α (B), and 2000 ng/ml LPS respectively (C). (D) Score-plot of the LDA model classifying different stimuli/concentrations. (E) Confusion matrix of 5-fold cross validation results. Numbers indicate the fraction of samples that is predicted correctly.

The discriminatory performance of the assay was validated on cultured HUVECs stimulated with different concentrations of either TNF-α or LPS. Upon stimulation with TNF-α or LPS, it was visually observed that HUVECs markedly changed their morphology (Figure 2A–C). These morphological changes included increased F-actin stress fibers and disruption of the VE-Cadherin adherence junctions upon stimulation with either TNF-α or LPS, and an elongated morphology upon TNF-α stimulation.

Manual parameter optimization of the automated image segmentation resulted in excellent segmentation of the images into individual cells (Figure A2). Principal Component Analysis (PCA) of the computed morphological fingerprints (Figure A3), showed clear clustering of replicate wells in the first 2 principal components (accounting for 53% of total variance). Remarkably, distinct trajectories could be observed for increasing concentrations of each stimulus, indicating a different morphological response of the cells to TNF-α compared to LPS.

To assess the discriminatory potential of the morphological fingerprints, an LDA classifier was trained and cross-validated using the different stimulants/concentrations as classes. Close grouping of technical replicates and different trajectories for each individual stimulant were observed in the score-plot (Figure 2D). Repeated 5-fold cross-validation by leaving out replicates and subsequent prediction of the stimulant/concentration of the left-out replicates showed a high degree of prediction accuracy of all TNF-α concentrations, with perfect prediction accuracy of TNF-α concentrations above 0.01 ng/ml (Figure 2E). LPS concentrations above 20 ng/ml were predicted with 100% accuracy, however lower LPS concentrations could only marginally be discerned from unstimulated controls.

### Endothelial Cell Morphology Correlates With the Clinical Stage of Liver Cirrhosis

**Figure 3:**
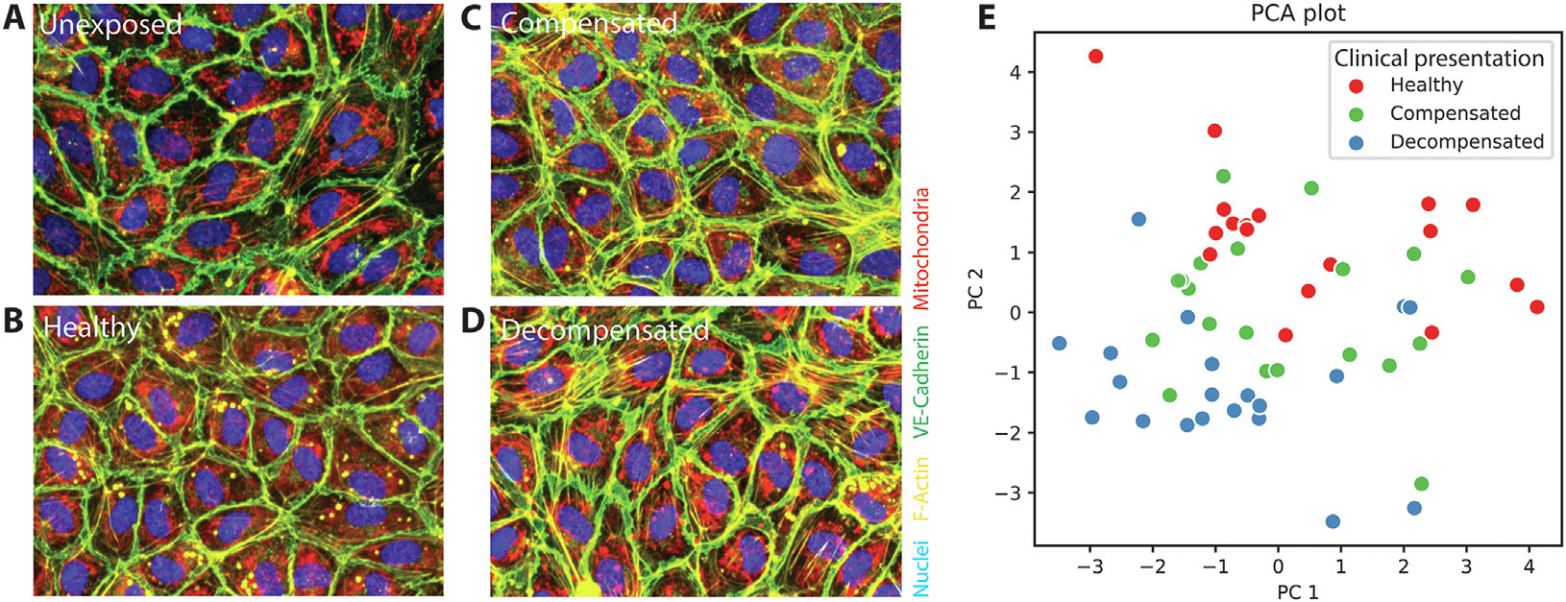
PCA plot of the morphological fingerprints of all patients per group. (A–D) Representative images of endothelial cells exposed to plasma from either unexposed control (A), healthy control (B), compensated patients (C) and decompensated patients (D). (E) PCA analysis (capturing 34% of total variance in PC1, and 23% in PC2) of all patient-specific morphological fingerprints.

Exposure of HUVECs monolayers to patient-derived plasma resulted in visually identifiable differences in morphology between cells exposed to plasma from healthy control subjects and cells exposed to plasma from decompensated patients (Figure 3A–D). Increased activation of the ECs was observed in ECs exposed to plasma from liver cirrhosis patients, when compared to healthy controls, most notably the increased F-actin stress fiber formation and irregular VE-Cadherin borders. ECs exposed to decompensated liver cirrhosis patient plasma displayed highly irregular VE-cadherin borders and differences in mitochondrial morphology, when compared to ECs exposed to plasma from compensated patients. Some residual platelets were observed as bright dots in the F-actin channel. Compared to unexposed controls (Figure 3A), cells exposed to plasma showed more F-actin throughout the cell, but less cortical actin.

Principal Component Analysis (PCA) analysis of the morphological fingerprints (normalized (Figure A9) and computed for each patient or healthy donor) showed separate clusters of patients with decompensated cirrhosis and of HCs (Figure 3E). Patients with compensated cirrhosis showed a tendency towards an intermediate profile between decompensated patients and HCs, as an overlap with both groups was observed.

To assess confounding effects of etiology, immunosuppressant use, and steroids use, PCA analysis and subsequent a cross-validated model-building approach was employed. Regarding etiology, only within the decompensated group slight grouping could be observed for alcohol related liver cirrhosis (Figure A4). However, no adequately performing model could be computed based on etiology (Figure A5). Also, no clustering or outlying datapoints were observed in the PCA score-plot for immunosuppressant therapy or steroid use (Figure A4). Therefore, none of these factors were accounted for in the subsequent analyses.

### Child-Pugh (CP) Class C Displays a Distinct Endothelial Cell Morphological Profile

**Figure 4:**
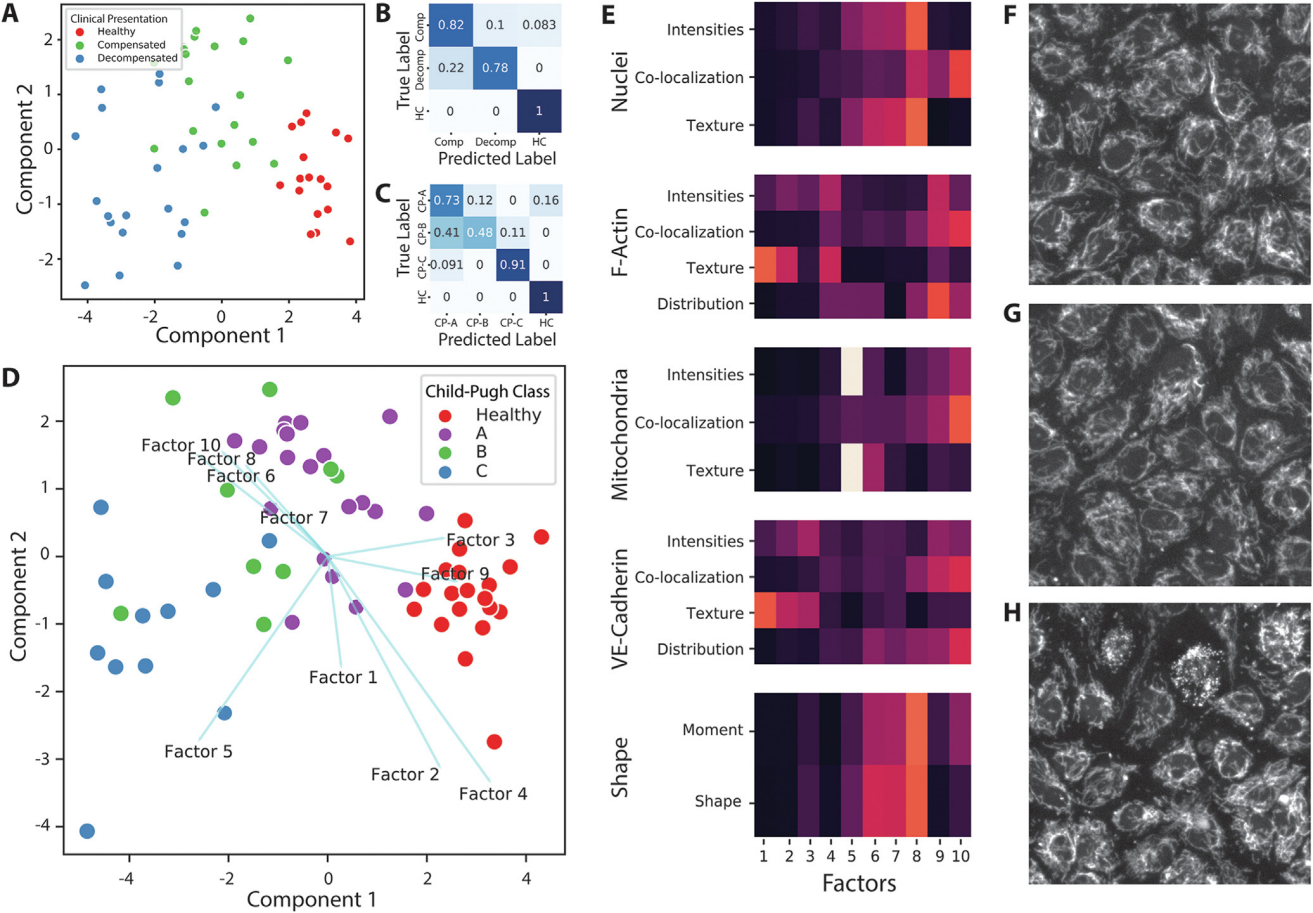
Supervised multivariate analysis of patient morphological profiles. (A) Score plot of the LDA classifying clinical presentation; healthy, compensated, and decompensated. (B) 10-fold cross validation results displayed as normalized confusion matrix of model classifying healthy controls, compensated, and decompensated liver cirrhosis. (C) 10-fold cross validation results displayed as normalized confusion matrix of model classifying healthy controls, and CP classes. (D) score plot of the LDA model colored according to Child Pugh (CP) class and healthy controls. Coefficients and direction for each factor are displayed in the same plot. (E) Contribution of cellular compartment and feature class to each Factor. Lighter colors indicate a higher contribution to the factor. Values were normalized by their total sum over all Factors. (F–H) Representative images of mitochondrial staining from ECs exposed to plasma from either healthy controls, CP class A/B, and CP class C liver cirrhosis patients, respectively.

Next, a supervised data analysis approach was employed to separate profiles based on the clinical stage (compensated cirrhosis, decompensated cirrhosis or HC), Figure 4A. After fitting of the data, some overlap was observed between the different clinical stages. Cross-validation results showed that the clinical stage could be correctly predicted from the morphological profile in the majority of cases, with some overlap of compensated cirrhosis with the other 2 groups (Figure 4B).

Next, we aimed to investigate whether the overlap between compensated and decompensated patients could be explained by disease severity expressed as CP class.^31^ To further investigate clustering by CP class a LDA model was fitted for the following classification: CP-A, CP-B, CP-C, and HCs (Figure 4D). A clear separation of CP-C from CP-A and CP-B was observed. The model also showed tight clustering of the HCs. Between CP-A and CP-B, a large overlap was observed, indicating that the morphological fingerprints of CP-A and CP-B are very similar. Cross-validation showed that the model was able to predict HCs and CP-C patients with high accuracy. Child-Pugh A (CP-A) and CP-B patients could be distinguished from other groups with high prediction accuracy, but not from each other (Figure 4C). In addition, PLS regression analysis was performed to observe if MELD score and CP score could be predicted from the morphological profiles. Both MELD score (R^2^ = 0.31 ± 0.26) and CP score (R^2^ = 0.33 ± 0.44) could be predicted from the morphological fingerprints with moderate accuracy (Figure A6).

### Mitochondrial Morphology Distinguishes Child-Pugh (CP) Class C From Child-Pugh (CP) Class A and B

The LDA model classifying CP classes was selected for further analysis into biological meaning. To facilitate interpretation of this model, model coefficients were visualized in the score plot as vectors for each factor (Figure 4D). The direction of each factor allows to assess their contribution to the class separation: factors oriented perpendicular to the line separating 2 groups are important for that separation. In our model, directions of factors 1, 2, 4 and 6, 7, 8, 10 were oriented perpendicular to the separation line between the clusters HC and CP-A/CP-B, indicating their importance in separation of these groups. Factor 5 was the only factor specifically directing towards the CP-C cluster, and nearly perpendicular to the separating line between the CP-C cluster and the CP-A/CP-B cluster. This indicates that factor 5, and to a lesser degree factor 3 and 9 are responsible for the distinction between the CP-C cluster and the CP-A/CP-B cluster. The CP-C cluster was orientated at an offset to the CP-A/CP-B cluster, orthogonal to the Factors 1, 2, 4 and 6, 7, 8, 10. Consequently, these factors do not contribute to the discrimination between the CP-C and CP-A/CP-B clusters, and these clusters are indistinguishable by these factors alone.

Since factor analysis was used for dimension reduction it is possible to visualize the construction of each fFactor in a heat-map (Figure 4E). By exploring this chart, mitochondrial morphology and intensity were found to be most discriminatory for separating CP-C from CP-A/CP-B (Factor 5), together with VE-Cadherin intensity and texture to a lesser degree (Factor 3 + 9). Factors responsible for separating HCs from cirrhotic patients consisted mostly of cell-shape, F-actin texture and distribution, and VE-Cadherin intensity and texture.

Above findings were validated by visually comparing the original images (Figure 4F–H). Indeed, mitochondrial morphology was found to be vastly different in ECs exposed to plasma from CP-C patients compared to ECs exposed to plasma from patients with CP-A, CP-B or HCs. Within a monolayer of ECs exposed to plasma from CP-C patients a large heterogeneity was observed in mitochondrial morphology, ranging from a phenotype comparable to CP-A and CP-B patients, to highly stressed phenotypes.

To investigate whether the observed clustering or changes in mitochondrial morphology correlated with presence of LPS in the samples, LPS concentrations in the plasma samples were measured and color-coded in the model score-plot (Figure A8). When comparing CP-C patients to HCs, a significant higher concentration of LPS was found in the samples (*P* < .001, Figure A6). However, this finding was not specific as presence of LPS was also observed in samples from patients with CP-A, CP-B, and HCs. In addition, no clear effect of LPS presence could be observed in the model clusters, as samples high in LPS showed no deviation from the assigned group. Additionally, no trend could be observed for CP-C patients regarding LPS concentration in the model score-plot (Figure A8).

## Discussion

In this study, we presented a novel *ex vivo* high-throughput, high-content, image-based screening assay for the assessment of morphological responses of ECs to patient-derived plasma. As a proof of concept we showed that the assay is capable of stratifying patients with liver cirrhosis according to disease severity. Moreover, we showed that it is possible to obtain biologically interpretable information from this stratification by combining factor analysis with linear discriminant analysis. In our cohort, we observed that mitochondrial changes are the most important characteristic for differentiating CP-C patients from patients with CP-A, CP-B, and HCs.

Image-based morphological profiling has been well established in the field of high-content screening. It has been widely implemented to investigate the effect of compounds on specific cellular processes such as the cell-cycle or translocation, identify gene and allele function, screening of small-molecule pathway regulators, or to investigate multiparametric morphological responses in compound library screenings.^24^^,^^42^^,^^43^ However, the implementation of image-based morphological profiling in the stratification of patients is novel.

Endothelial cells (ECs) are ideally suited for image-based screenings, since dramatic morphological changes are observed in response to inflammatory and other stimuli.^26^, ^27^, ^28^ In order to be able to differentiate best between EC phenotypes and states, a panel of morphological markers was selected to capture most information on EC phenotype. Beside staining for the nucleus, the mitochondria, and F-actin, we also included a staining of VE-cadherin (instead of the endoplasmic reticulum staining in the original publication^25^) to provide functional information on EC barrier function, as the morphology of adherence-junction provides functional information on endothelial barrier integrity.^44^^,^^45^

To validate the ability of the assay to capture and differentiate between subtly different EC morphologies, we validated our analysis pipeline using examples of ECs exposed to different concentrations of the liver cirrhosis associated proinflammatory stimuli TNF-α and LPS. Stimulus and dose dependent morphological features were well represented in the created profiles, as 2 distinct ordered trajectories of increasing concentrations TNF-α or LPS were observed. Importantly, these trajectories represent 55% of total variance, indicating most information captured in the morphological fingerprints reflects morphological changes induced by the stimulant exposure.

Supervised analysis was used to further assess the quality of the extracted profiles. The correct concentration and stimulus could be predicted from the morphological fingerprints with excellent accuracy for higher concentrations of TNF-α or LPS, confirming the high discriminatory ability and stability of these profiles. Furthermore, the sometimes subtle differences between ECs stimulated with lower dose TNF-α or LPS were well-represented in the profiles, as even lower concentrations could be adequately predicted. However, ECs exposed to the lowest LPS concentrations resembled unexposed ECs to a large degree, and thus could not be distinguished from each other or unexposed controls.

Given forementioned results, we were convinced that the phenotypical information captured in the profiles would be sufficient to stratify patient-specific EC morphology induced by *ex vivo* exposure to patient derived plasma.

Exposure of ECs to patient-derived plasma resulted in visible morphological differences between patients with compensated cirrhosis, decompensated cirrhosis and HCs. Discriminatory features between these clinical groups were well represented in the dataset, as clinical presentation was the main observed effect in the PCA model, accounting for 57% of total variance. A large degree of separation between decompensated patients and healthy controls was observed in the PCA model. Moreover, clustering of compensated patients was located in the middle of these 2 groups with overlap with both groups, suggesting compensated patients to have a more intermediate profile.

Further interrogation of the profiles by supervised analysis showed a clear overlap between compensated and decompensated patients. Therefore, we further investigated this overlap by modeling the clinical CP class as a severity measure. Interestingly, patients with CP class C seemed to have a distinct profile from CP classes A and B, while the morphological profiles of CP class A and B seemed to be similar. This was also confirmed by cross-validation, where CP-C could be predicted from the morphological profiles with 90% accuracy, but CP-A and CP-B could not be distinguished from each other.

Actin organization, cell shape, and VE-Cadherin morphology were identified to be most important for differentiating patients with liver cirrhosis from HCs. It has been well described that the formation of F-actin stress fibers, and VE-Cadherin reorganization are clear indications of EC activation.^26^^,^^28^ Therefore, our results strongly suggests that the extent of EC activation correlates with the severity of the liver cirrhosis.

The most important feature responsible for the CP-C distinct profile was a changed mitochondrial morphology, showing a shift towards a phenotype resembling mitochondrial fission. A shift towards the mitochondrial fission phenotype has previously been described in ECs for a number of other systemic diseases including cardiovascular diseases and diabetes mellitus.^46^ Moreover, a mitochondrial fission phenotype has also been described in TNF-α or LPS stimulated cultured ECs, where mitochondrial fission is thought to mediate the EC inflammation response by contributing to sustained NF-κB activation.^47^^,^^48^ In liver cirrhosis, signs of mitochondrial dysfunction have recently been described, especially in relation to progression towards multiorgan failure.^49^^,^^50^ Unfortunately, ECs were not investigated in these studies. Intriguingly, the shift in mitochondrial morphology is observed in addition to the EC activation which is found in almost all liver cirrhosis patients. Therefore, it is tempting to speculate that mitochondrial dysfunction upon EC activation results in a shift towards a severe stage of decompensated cirrhosis and that this mitochondrial dysfunction may play a role in sustaining NF-κB activation. Further functional investigation would be needed to confirm this hypothesis.

Systemic inflammation is well-recognized in decompensated cirrhosis.^11^, ^12^, ^13^ Proinflammatory and anti-inflammatory cytokines that act upon ECs such as vascular endothelial growth factor, TNF-α, interleukin-6, interleukin-8, and interleukin-1ra, were found to be significantly higher in the peripheral plasma of patients with decompensated cirrhosis compared to HCs.^13^ The severity of EC activation, and thus morphological responses, are determined by the balance between these pro-, and anti-inflammatory signals and integration thereof by complex signaling pathways.^51^ Our data show that peripheral plasma from cirrhotic patients indeed induce EC activation, and that the degree of EC activation is correlated with the severity of the disease. Therefore, the results of this assay indicate that systemic endothelium activation in response to circulation plasma factors may contribute to the multisystem inflammatory state associated with the several stages of liver cirrhosis. These findings also support the notion that the progressive systemic inflammatory process is a main driver behind organ hypoperfusion and cell dysfunction in the pathogenesis of cirrhosis.^52^

Severe EC dysfunction, resulting from bacterial-derived LPS and host-derived factors including cytokines, activated platelets leukocytes and damage-associated molecular patterns, has been identified to be the key factor in systemic inflammatory response syndrome and multiple organ failure in sepsis.^53^^,^^54^ Our results show highly activated ECs, especially in CP-C patients, suggesting EC dysfunction might play a similar role in liver cirrhosis pathology. Interestingly, in our dataset, LPS concentration by itself seemed not to determine the EC morphological response, as no trends correlated to LPS concentrations were observed. Moreover, HC samples positive for LPS, likely resulting from contamination, showed no severely affected phenotype and clustered together with other HCs, further indication morphological responses were not derived from LPS stimulation.

The focus of this study was to develop the assay and to perform a pilot in a patient cohort to prove its functionality. For this reason, the sample size was small, groups tended to be not completely comparable (for example distribution of etiology and age), and model prediction results might be overly optimistic. Although no effect of etiology on the clustering was observed, further analysis in larger cohorts is needed to confirm current findings. Moreover, the assay in its current form only serves for patient stratification, and by identification of important cellular features for the stratification is hypothesis generating. To decipher the underlying mechanisms, subsequent analyses are still required. However, the high-throughput screening nature of the assay makes it possible to identify factors in the plasma responsible for the EC activation by inhibitor library screenings. Future efforts will therefore be directed toward screening of a large cohort of liver cirrhosis patients, with the aim to predict disease outcome and progression, and identification of the responsible circulating plasma factors. Once established, our platform technology may also serve to diagnose disease progression and the monitoring of disease interventions such as the suppletion of albumin or the effective use of antibiotics in cirrhosis patients with infections.

## Conclusion

We present a novel high throughput, *ex vivo* morphological profiling assay for the assessment of endothelial cell responses to patient-derived plasma. To the authors knowledge, this is the first time the *“cell painting assay”* has been utilized to quantify EC responses to patient-derived plasma in order to stratify patients. We demonstrated the usability of this assay to stratify patients based on EC morphology, to correlate between EC phenotype to disease severity, and in the identification of important cellular features relevant to disease pathophysiology. Taken together, this assay might be a valuable tool for the generation of new hypotheses in the study of systemic diseases. The ability of this assay to translate patient-specific EC morphological responses into one single data vector, rich in information, makes it a powerful new tool for future use in the prognostication of disease progression and the prediction of patient-specific therapeutic responses, thereby contributing to the development of personalized medicine.

## Supplementary Materials

Figures A1–A9
