# Supplementary material for: Novel Morphological Profiling Assay Connects ex Vivo Endothelial Cell Responses to Disease Severity in Liver Cirrhosis
Source: Gastro Hep Adv. 2023 Oct 24;3(2):238–49. doi: 10.1016/j.gastha.2023.10.006 (PMC11307659; doi:10.1016/j.gastha.2023.10.006)
Supplement: Figures A1–A9 [file mmc1.docx]

# SUPPLEMENTARY MATERIALS

## Figure S1

**
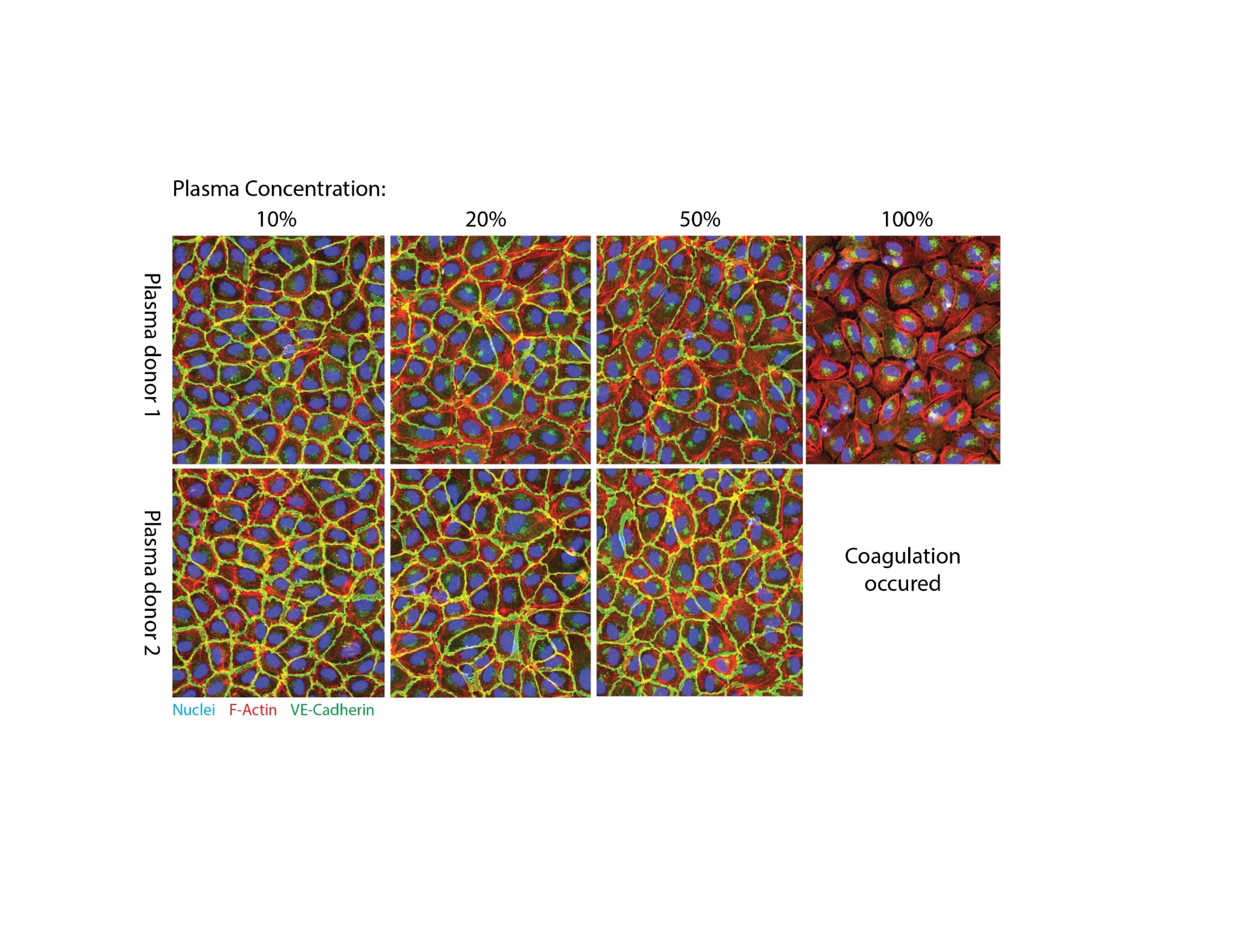
**

**Supplemental figure 1:** Examples of single donor Human Umbilical Vein Endothelial Cell morphology after exposure to different concentrations(10%, 20%, 50%, and 100%) of plasma derived from healthy controls.

## Figure S2

**
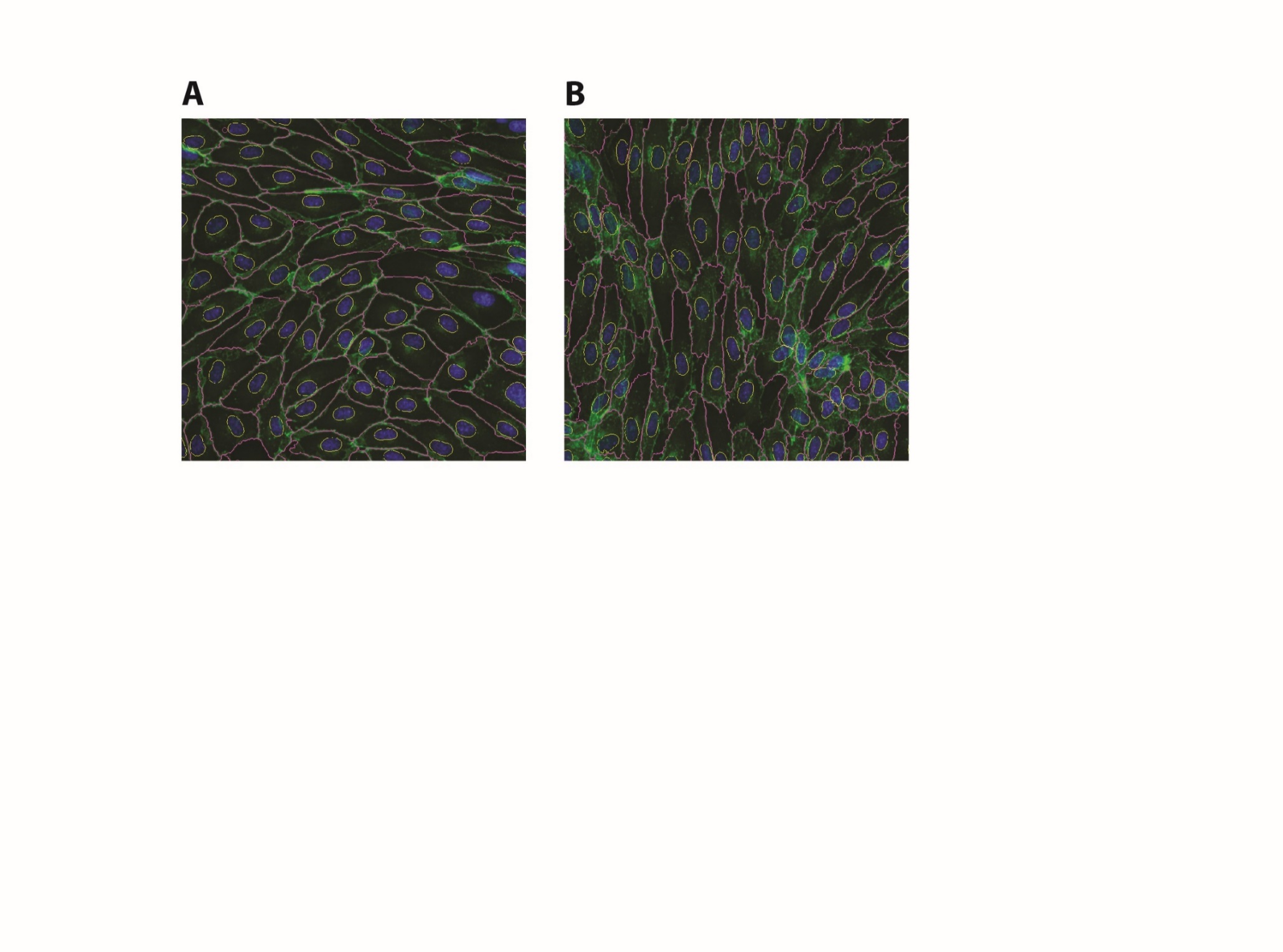
**

**Supplemental figure 2: Automated tracing of the nuclei and cell-borders**

Overlay showing automated tracing of the nuclei (yellow) and cell-borders (purple) for endothelial cell (EC) monolayers exposed to 0,1 ng/ml (A) and 1.0 ng/ml (B) TNF-α. The algorithm traces the VE-Cadherin adherens junctions (green) for identification of cell borders.

## Figure S3


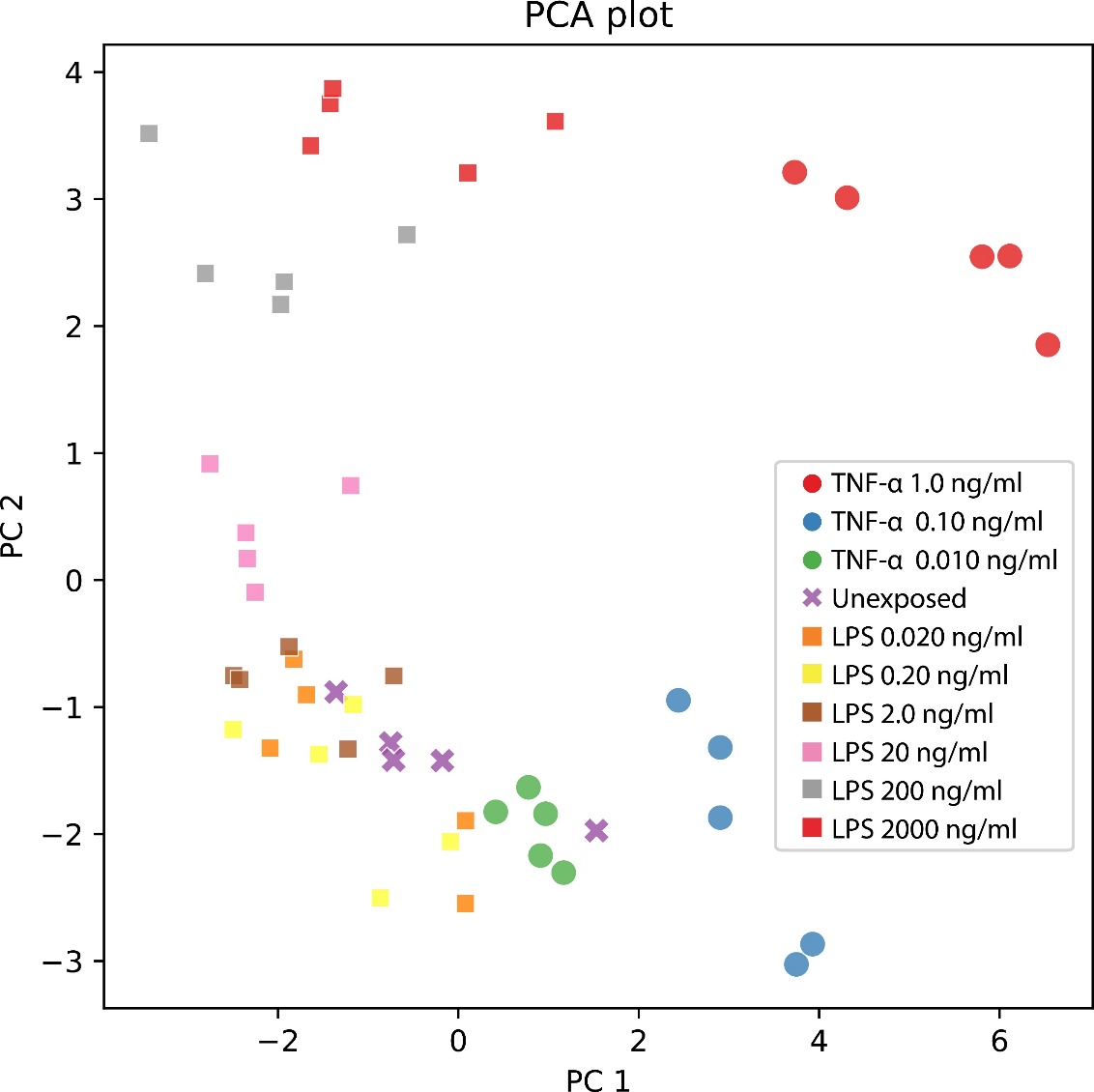


**Supplemental figure 3:** PCA(capturing 31% and 22% of variance in PC1 and PC2 respectively) score-plot of morphological fingerprints of HUVECs stimulated with different concentrations TNF-α or LPS.

## Figure S4


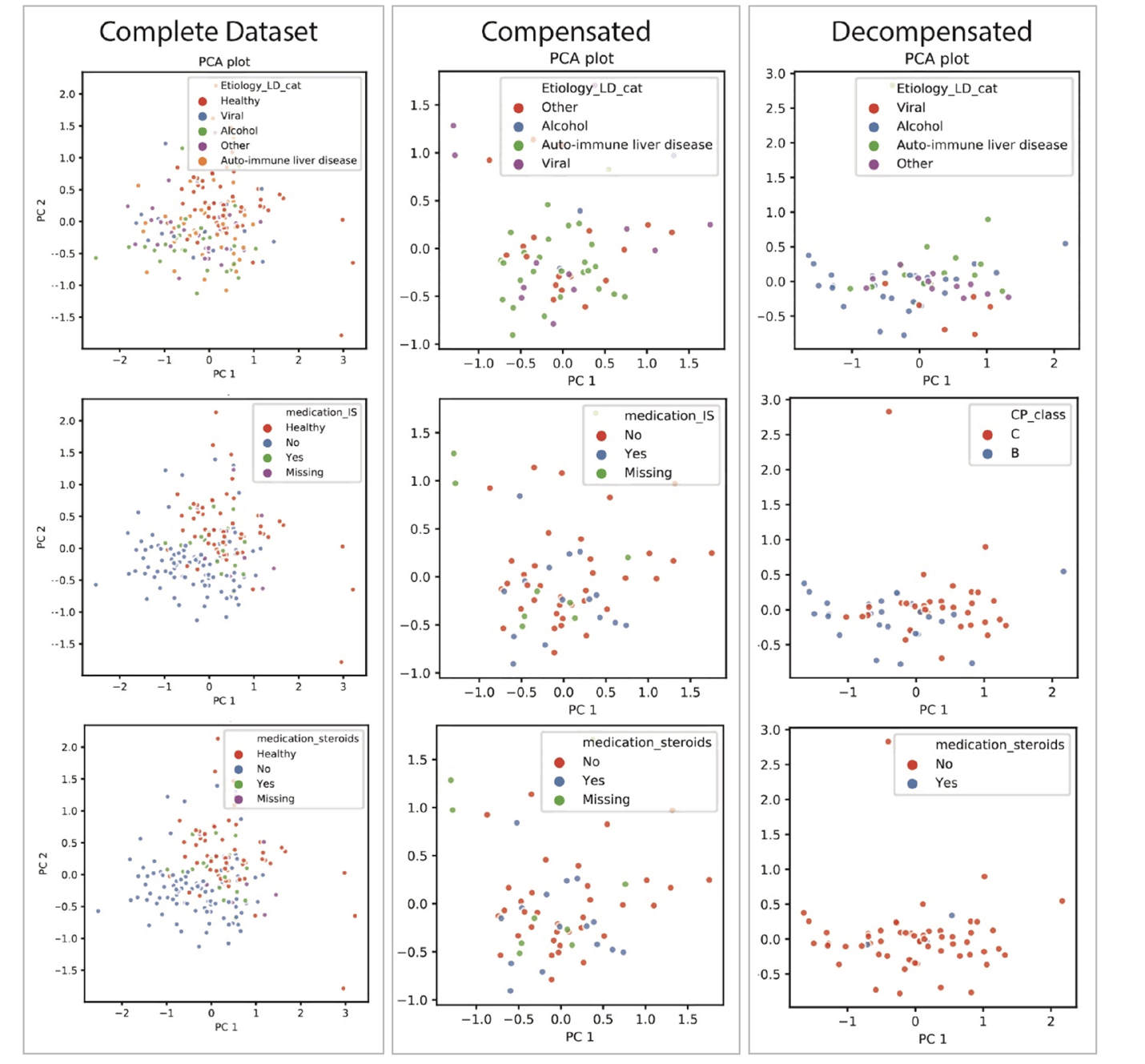


**Supplemental figure 4: PCA plots of the complete dataset containing all technical replicates** PCA score-plots of the complete dataset, compensated group, and decompensated group, color-coded for etiology, immunosuppressant use, or steroid use, respectively.

## Figure S5


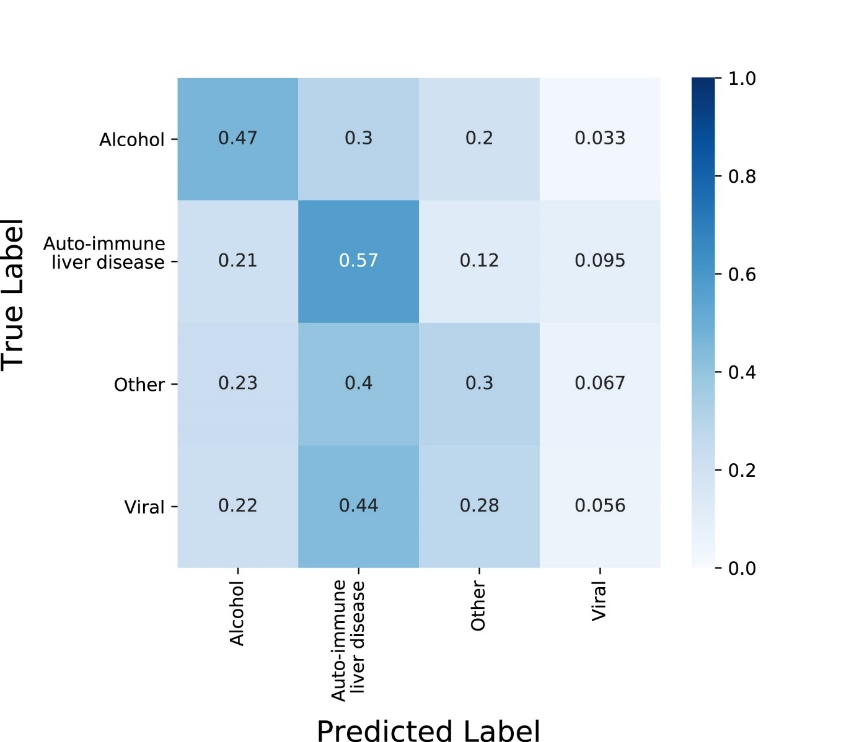


**Supplemental figure 5:** Cross-validation results of the LDA model predicting etiology, displayed in a confusion matrix.

## Figure S6


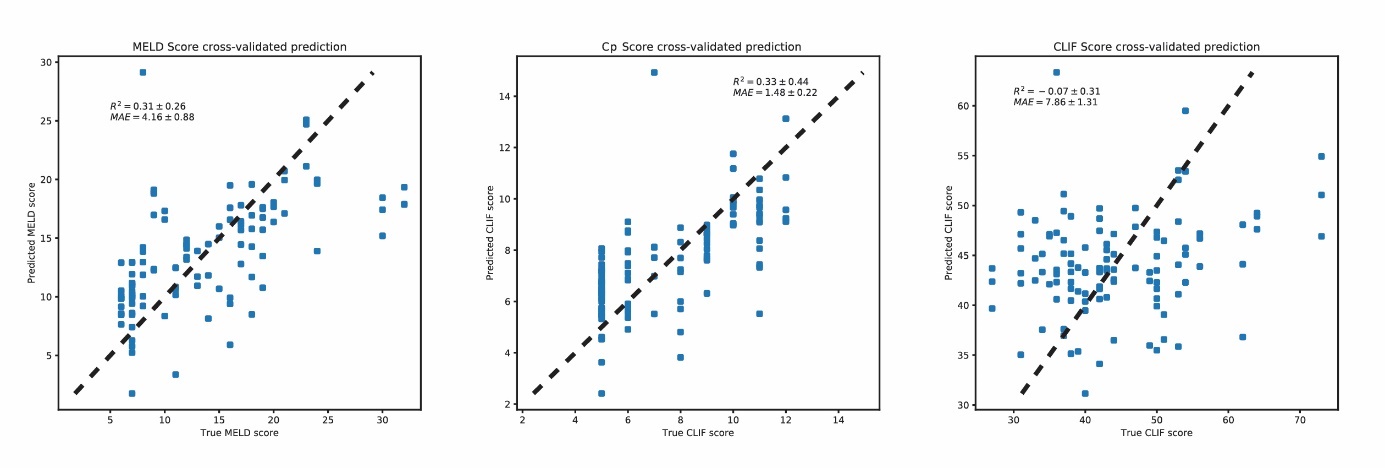


**Supplemental figure 6:** 10-fold cross-validation results of two different PLS models predicting either MELD score or Child-Pugh score. Predicted score by the model is plotted against the true score in order to visualize deviation from the perfect model and to observe for non-linearity.

## Figure S7


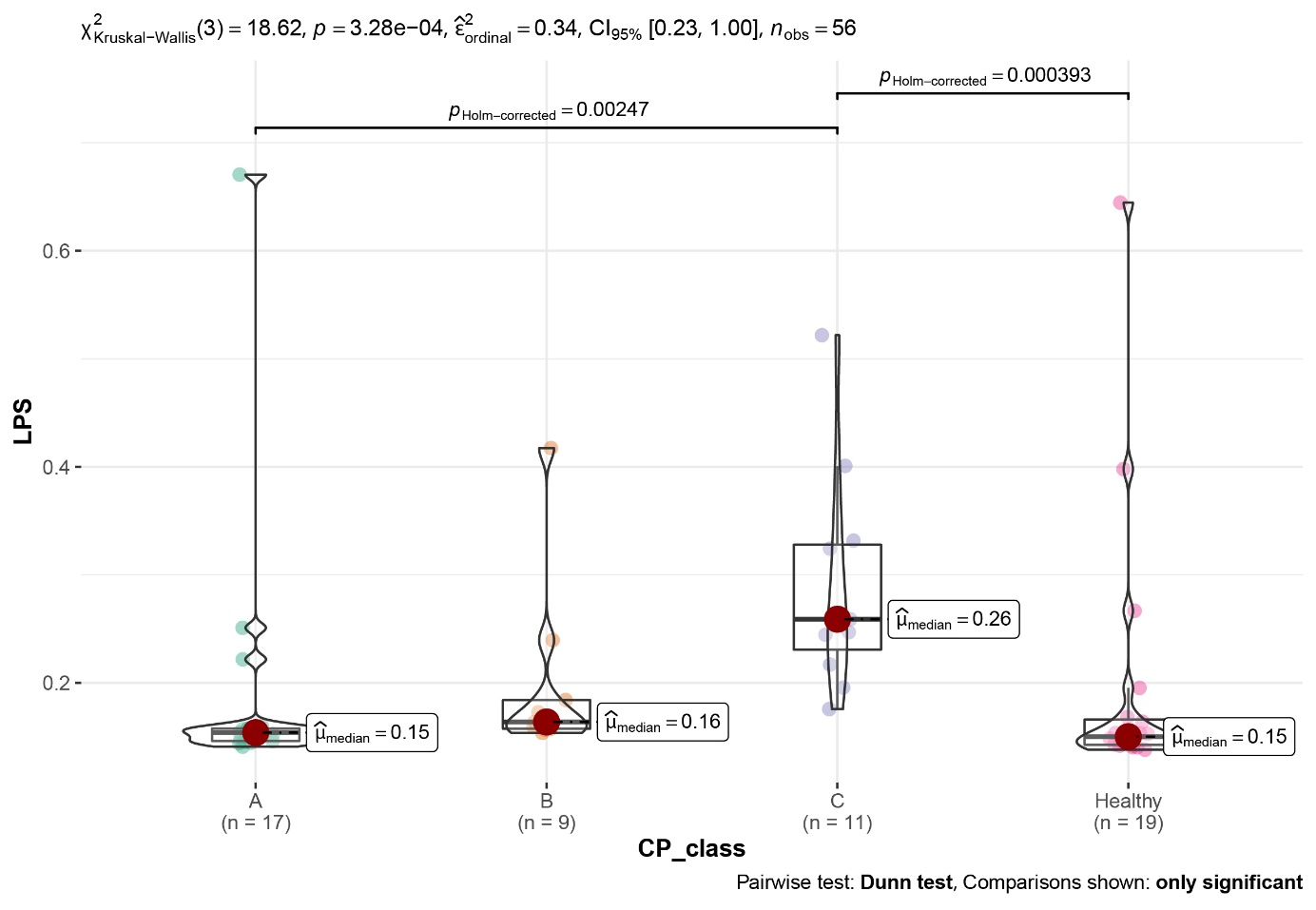


**Supplemental figure 7:** Analysis of the relative LPS concentrations in the samples.

## Figure S8


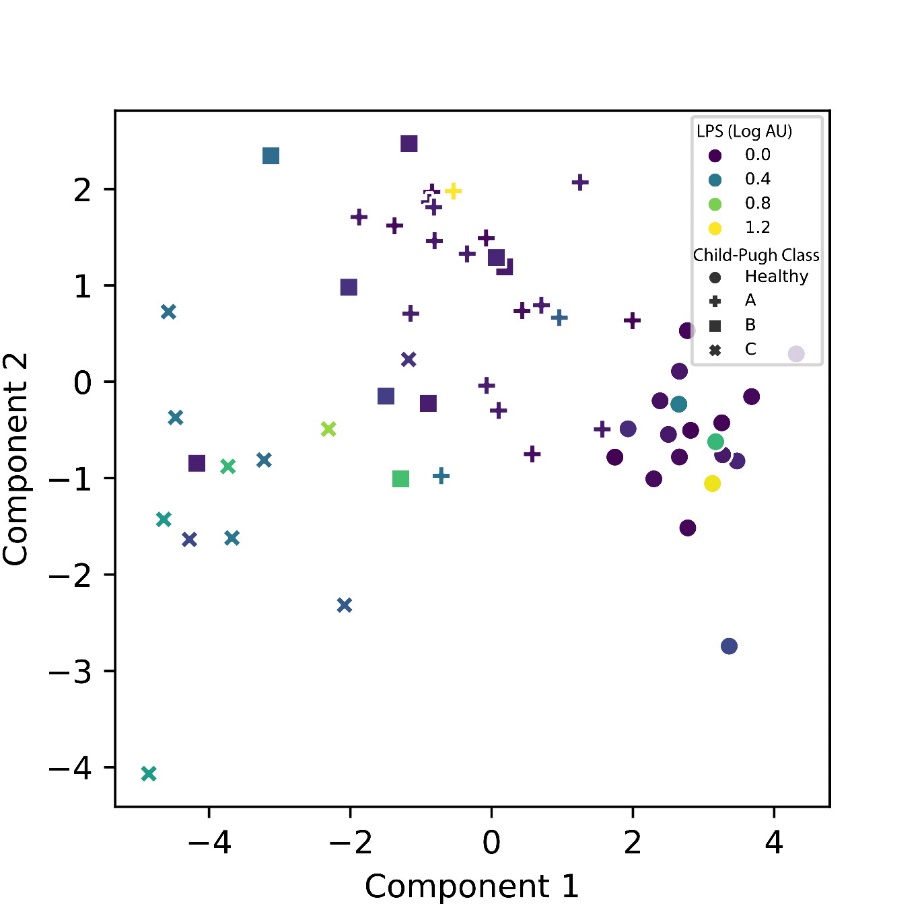


**Supplemental figure 8:** The score-plot of the LDA model classifying Child-Pugh class (different CP classes are displayed as different symbols) colorcoded according to LOG LPS concentrations. Lighter, more yellowish colors indicate higher LPS concentrations (the LOG scale was chosen for better visualization).

## Figure S9


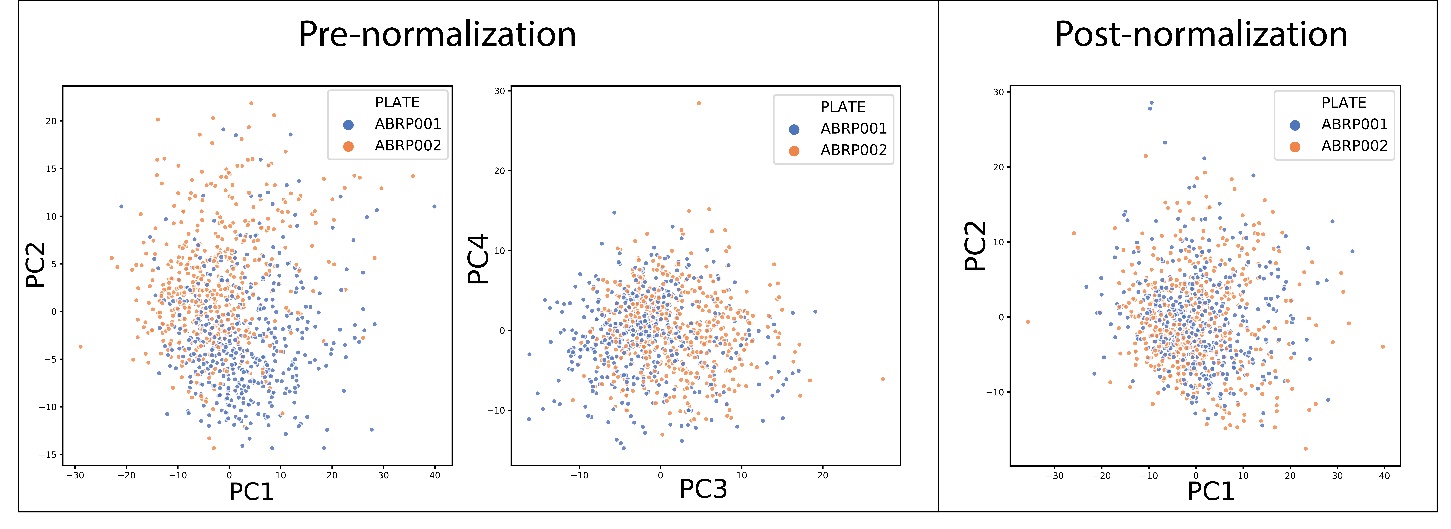


**Supplemental figure 9:** PCA analysis of high-dimensional single-cell profiles before and after normalization. Deviation between the two plates is present in the first three principal components, accounting for 37% of total variance.
